# Supplementary material for: Learning a reactive potential for silica-water through uncertainty attribution
Source: Nat Commun. 2024 Jul 17;15:6030. doi: 10.1038/s41467-024-50407-9 (PMC11254924; doi:10.1038/s41467-024-50407-9)
Supplement: Supplementary file 1 — Supplementary Information [file 41467_2024_50407_MOESM1_ESM.pdf]

Supplementary Information for: Learning a reactive potential for silica-water through uncertainty attribution

Swagata Roy[1], Johannes P. Dürholt[2], Thomas S. Asche[2], Federico Zipoli[3] and Rafael Gómez-Bombarelli[1,*]

[1]Department of Materials Science and Engineering, Massachusetts Institute of Technology, Cambridge, MA, USA

[2]Evonik Operations GmbH, Essen, North Rhine-Westphalia, Germany

[3]IBM Research Europe, Saümerstrasse 4, 8803, Rüschlikon, Switzerland

[*]Corresponding Author. E-mail: rafagb@mit.edu

1 Structural properties of α-Quartz

Property	NNIP	Exp	DFT	
			GGA	LDA
Si-O	1.612	1.6137	1.6170	1.6045
O-Si-O	110.2	109.0	108.4	109.3
Si-O-Si	141.8	143.7	149.5	144.1

Supplementary Table 1: Structural properties bond lengths (in Å) and bond angles (in degrees), for α-quartz. NNIP: Neural Network inter-atomic potential, EXP: Experimental. LDA and GGA are two different DFT theories. The EXP and DFT data are obtained from literature [1]

We obtained different bond lengths and bond angles in alpha-quartz at room temperature and compared them to experimental and DFT data. The properties are predicted almost accurately. The results are shown in Supplementary Table 1.

2 Attribution vs bond length

Generally, we calculated attribution as per-atom uncertainty by taking a derivative of variance in force or energy to atom positions. Active learning based on this attribution was sufficient to improve our NNIP through generations. However, we also did a test on how attribution varies with bond lengths or inter-atomic distances. We chose a periodic cubic box of 100 water molecules and calculated attribution by taking a derivative of variance in predicted forces to inter-atomic distances instead of atom positions. We plotted the attribution vs inter-atomic distance in Supplementary Figure 1. We observed that O-H bond length around 1 Å shows more attribution than O-O and H-H bonds. This shows that different combinations of atom positions may lead to important information on uncertainty for active learning.

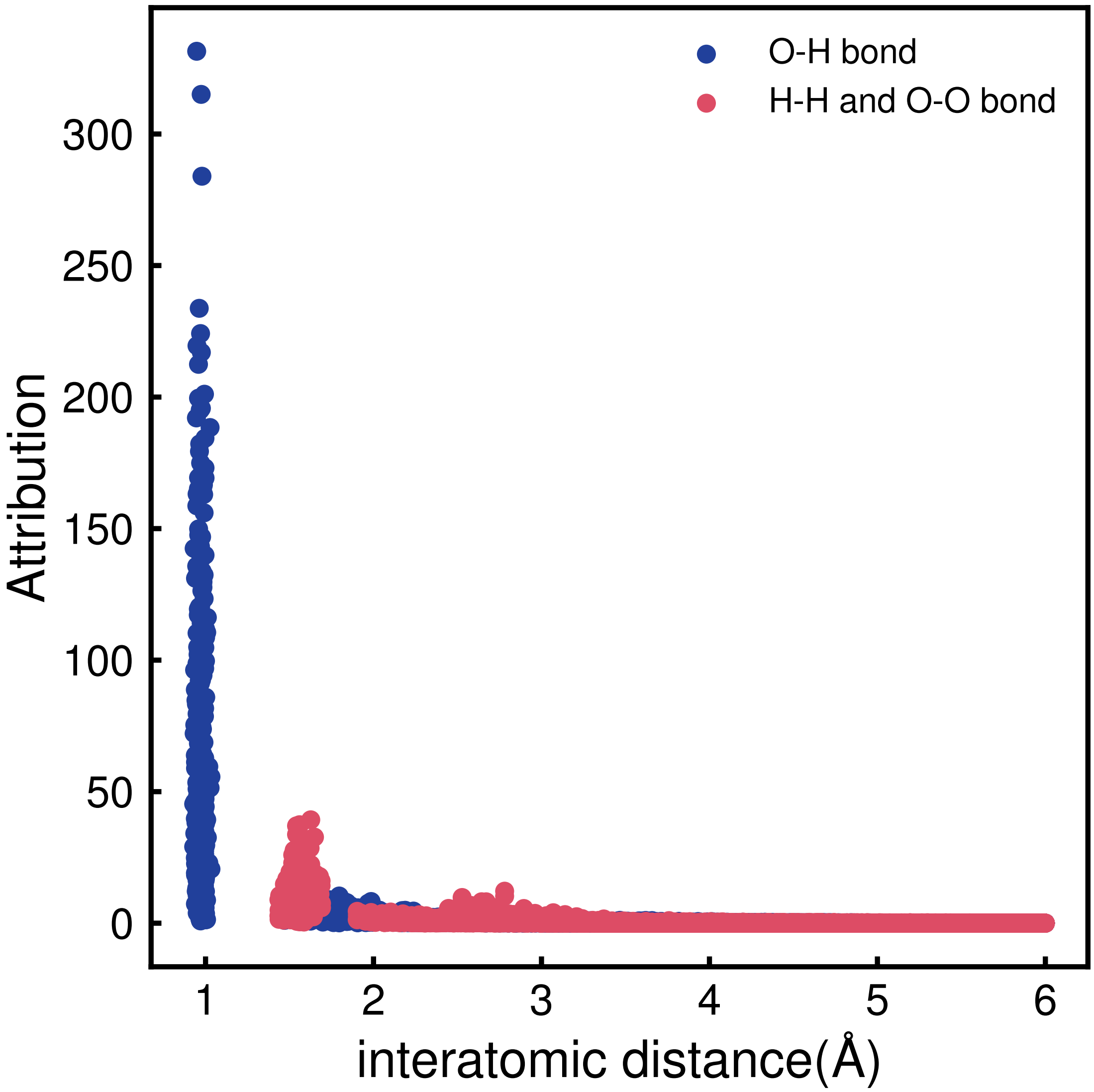

Supplementary Figure 1: Attribution on inter-atomic distances.

3 Free energy of S_N2 mechanism of dimerization

We plotted the free energy profile of S_N2 mechanism of silicate dimerization in a neutral medium in Supplementary Figure 2. The product with H_3O^+ and OH^- has 79 kJ/mole energy higher than the reactant and the reaction has an activation energy of 180 kJ/mol which is higher than the lateral mechanism. Thus the mechanism occurs through the lateral side attack mechanism.

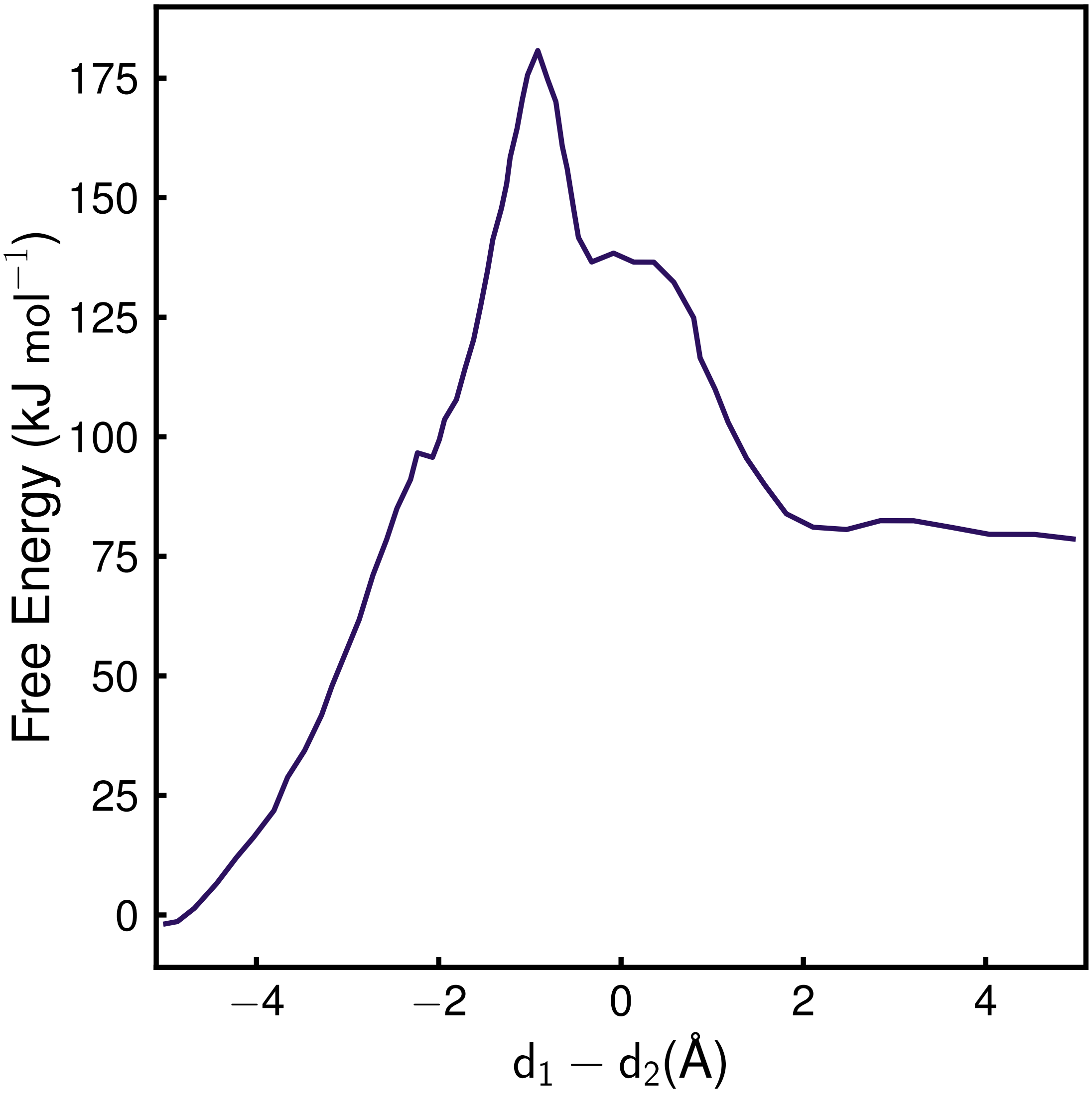

Supplementary Figure 2: Free energy profile of silicate dimerization with S_N2 mechanism

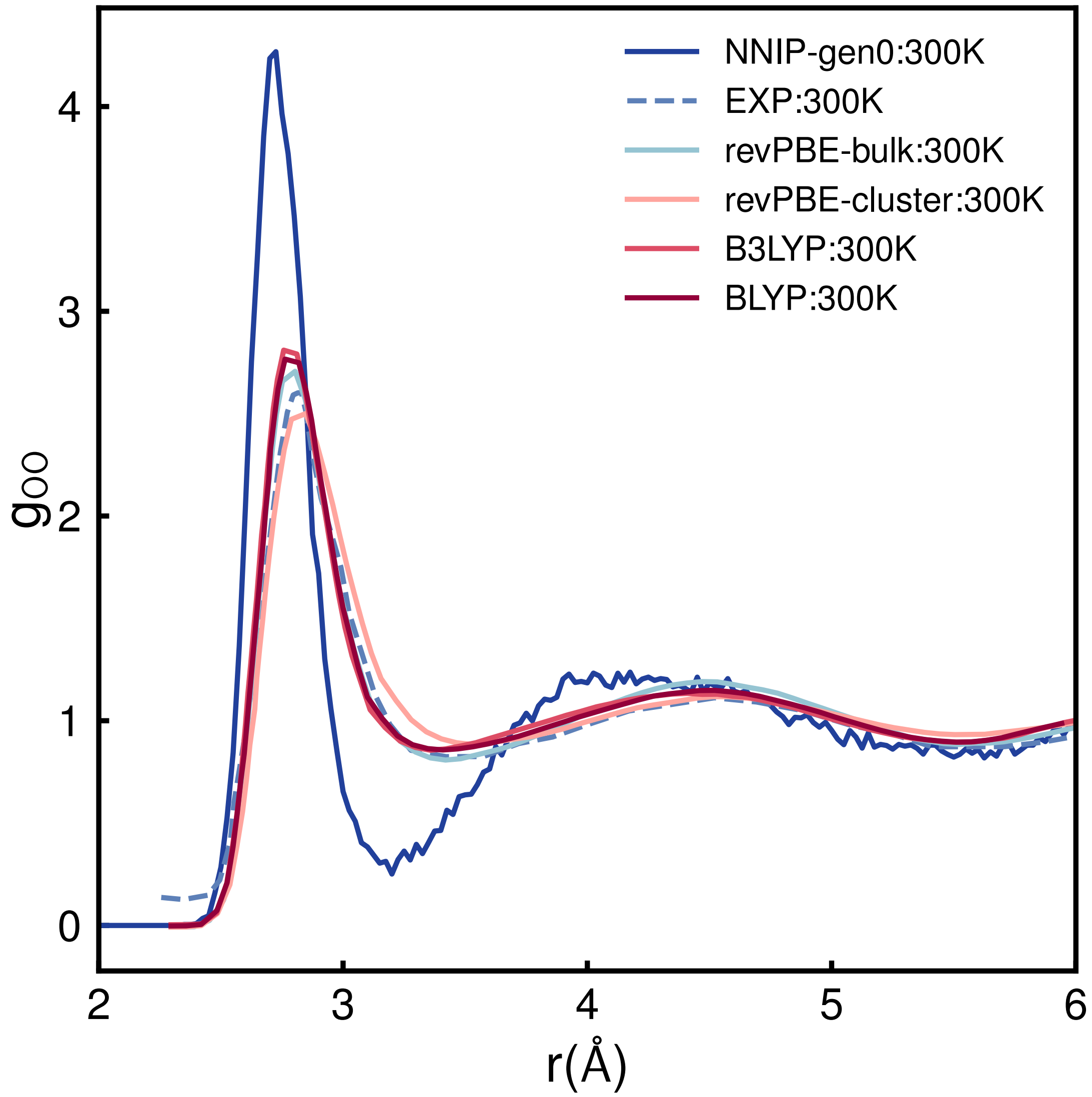

Supplementary Figure 3: O-O radial distribution function at 300 K calculated by constant-temperature, constant-volume ensemble (NVT) simulations with our base generation NN-based interatomic potential (NNIP) and compared to experimental and GM-NN potentials' results.NNIP-gen0: Neural network inter-atomic potential trained on preliminary dataset only; EXP: experimental data; revPBE-bulk: GM-NN potential trained on periodic water box calculated at the revPBE-D3 level of theory; revPBE-cluster: GM-NN potential trained on water clusters calculated at the revPBE-D3 level of theory; B3LYP: GM-NN potential trained on water clusters calculated at the B3YLP-D3 level of theory; BLYP: GM-NN potential trained on water clusters calculated at the BYLP-D3 level of theory. The base generation NNIP without active learning cannot produce the accurate O-O radial distribution function.

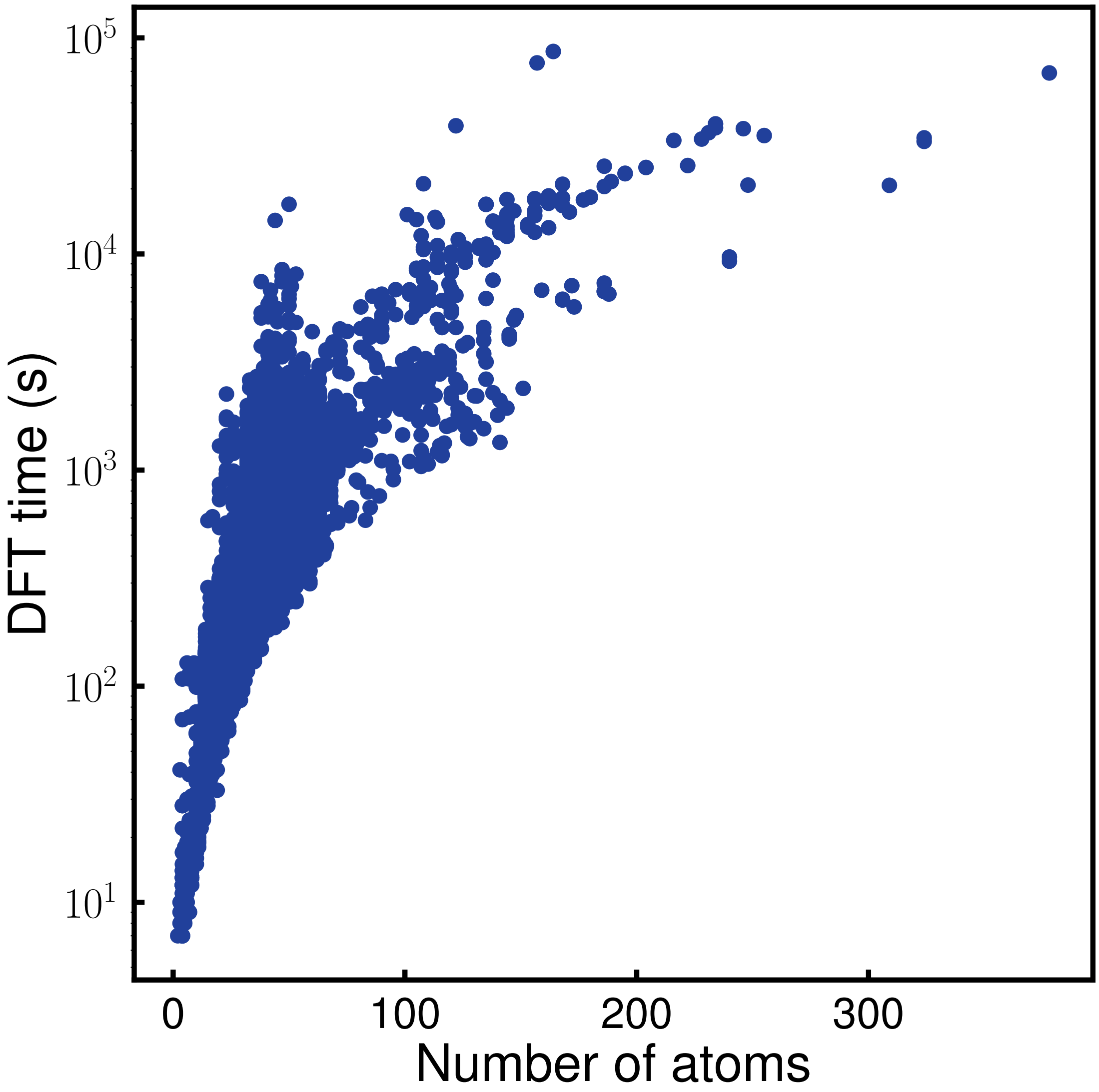

Supplementary Figure 4: Density Functional Theory (DFT) calculation time vs Number of atoms in the molecular clusters. We see the DFT time varies exponentially with the number of atoms. Attribution-based active learning helps limit the number of atoms in the molecular clusters within 300 atoms.

4 Supplementary References

1. Demuth, T., Jeanvoine, Y., Hafner, J. & Ángyán, J. G. Polymorphism in silica studied in the local density and generalized-gradient approximations. *Journal of Physics Condensed Matter* **11,** 3833–3874 (1999).
